# Minimally Invasive Robotic-Assisted Complex Adult Spinal Deformity Correction in a Surgical Specialty Hospital: Bringing Adult Spinal Deformity Care Closer to Home

**DOI:** 10.3390/jcm15082913

**Published:** 2026-04-11

**Authors:** Roland Kent

**Affiliations:** Axis Spine Center, Division of Northwest Specialty Hospital, 7600 N Mineral Dr, Ste 450, Coeur d’Alene, ID 83815, USA; r.kent@axisspinecenter.com

**Keywords:** robotic-assisted surgery, adult spinal deformity, minimally invasive spine surgery, surgical specialty hospital, robotic pedicle screw

## Abstract

**Background/Objectives**: Adult spinal deformity (ASD) correction is a complex surgery to restore spinal alignment and relieve patients’ symptoms. Modern techniques and technologies allow for aggressive surgical correction in tissue-friendly ways that preserve anatomy and may enable faster recovery. Robotic-assisted posterior spinal stabilization may be used as an adjunct to complex ASD reconstruction to facilitate a minimally invasive approach, reduce perioperative morbidity and physiological insult, and allow for the performance of procedures traditionally reserved for large academic centers to be effectively performed by qualified surgeons in optimized patients at smaller hospitals with fewer resources. The objective of this study is to assess realignment, perioperative complications, and patient-reported outcomes of complex, minimally invasive, robotic-assisted adult spinal deformity correction in a surgical specialty hospital. **Methods**: Demographic, surgical, and perioperative data were collected from the medical record. The Oswestry Disability Index (ODI) and Numeric Rating Scale (NRS) for pain scores were collected preoperatively and at regular post-op visits. X-rays were captured preoperatively before hospital discharge and at follow-up visits. **Results**: Fifty consecutive deformity patients were corrected with a two-stage approach (anterior column reconstruction followed by posterior stabilization with robotic-assisted screw placement on the next day) at a 48-bed (eight operating rooms), surgeon-owned, subspecialty hospital. The average patient age was 70 years, and 64% were female. The average estimated blood loss (EBL) values for the first and second stages were 62 mL and 205 mL, respectively. The average operative time was 172 min during the first stage and 210 min for the second stage. Three interbody spacers (first stage) and 16 screws (second stage) were inserted on average in each procedure. The average length of stay (LOS) in the hospital was 5 days, and the average follow-up period was 10.6 months. No patients required a transfer to another facility with intensive care unit (ICU) capabilities, and none required a revision of hardware placement. There was an average reduction in the lumbar coronal scoliotic curve of 14.5° and an increase in lumbar lordosis of 14.8° at the latest follow-up (*p* < 0.01). The average mismatch between pelvic incidence and lumbar lordosis (PI-LL) preoperatively was 17.6°, which was reduced to 9.6° at the latest postoperative follow-up (*p* < 0.01). Mean ODI (%) and NRS scores were significantly improved by 33.8% (46.7 ± 13.3 to 30.9 ± 19.8; *p* < 0.01) and 55% (6.0 ± 2.2 to 2.7 ± 2.6; *p* < 0.01), respectively, at last follow-up. **Conclusions**: This study demonstrates the feasibility of performing complex, robotic-assisted ASD corrective surgery in a surgical specialty hospital, achieving significant correction of sagittal and coronal deformities, relieving patients’ symptoms, and offering efficiency and consistency to pedicle screw placement. This study demonstrates that a minimally invasive approach to complex deformity reconstruction reduces perioperative morbidity with decreased operative times, EBL, and LOS when compared to historic controls. This approach allows for the democratization of deformity care in that procedures typically reserved for large academic centers can be successfully accomplished at smaller institutions in optimized patients by qualified surgeons with appropriate perioperative support staff.

## 1. Introduction

With the age of the U.S. population and number of fusion procedures performed increasing yearly, adult spinal deformity (ASD) is becoming more prevalent due to spinal degeneration and iatrogenic flatback [1]. Historical surgical treatment of moderate to severe ASD has involved open, multilevel spine fusion [2]; however, these surgeries are frequently associated with high rates of complications, need for reoperation, and extra procedural cost, especially in the elderly and/or those with multiple comorbidities. Within the 2-year postoperative period, major and overall complication rates have been reported to be 48% and 70%, respectively, with the majority of these complications related to implants and/or neurological issues [3,4].

Due to the increased risk involved, these procedures are traditionally performed at large academic institutions with multiple resources to care for complex patients. These hospitals are typically confined to large cities, which makes access to care for patients in more rural areas difficult. Patients from rural areas, having already traveled long distances for the index surgery, may have to travel those same distances again to address unforeseen postoperative complications.

Modern minimally invasive surgical (MIS) technologies for ASD correction can be used to mitigate the complications associated with traditional open access. Lower blood loss, quicker recovery, decreased postoperative analgesic and opioid requirements, lower complication rates, shorter operative times, and reduced length of hospital stays are reported advantages of MIS compared to open techniques [5,6,7]. MIS approaches commonly used in ASD corrective surgery are lateral lumbar interbody fusion (LLIF) and percutaneous screw placement [8]. By incorporating hyperlordotic spacers and anterior column reconstruction through anterior and lateral approaches, surgeons can correct the sagittal plane deformity without the need for extensive subtraction osteotomies [9,10].

The introduction of robot-assisted MIS surgery allows for accuracy, efficiency and consistency in the posterior stabilization stage of the corrective procedure, including placement of pedicle and S2 alar iliac screws and reduced radiation exposure during insertion of interbody and screw implants. Additionally, preoperative posterior construct planning allows for the virtual alignment of screw heads from level to level during a time when the patient is not yet under anesthesia and exposed to the dangers of surgical intervention. The use of robotic assistance to accurately and efficiently reproduce the planned construct facilitates rod passage and deformity correction while preserving surrounding soft tissues [11,12]. Recent systematic reviews have confirmed the accuracy and safety of robotically placed pedicle screws in ASD surgery [13] and have provided comprehensive overviews of the current state of robotic-assisted spine surgery, including its clinical outcomes, learning curves, and future directions [14]. Studies examining the learning curve of robotic platforms, including the ExcelsiusGPS^®^ system used in the present study, have demonstrated rapid proficiency development, with significant improvements in operative efficiency observed after approximately 14 to 25 cases [15]. Furthermore, preoperative robotic construct planning has been shown to facilitate the design and placement of complex multi-rod constructs in MIS ASD surgery, further enabling minimally invasive approaches to deformity correction [16].

The goal of the current study is to analyze perioperative data, assess early postoperative patient alignment, and examine early patient-reported outcomes after MIS robotic-assisted ASD correction surgery. We will compare these data to historic controls to make a case for the democratization of complex deformity care to nonacademic and resource-limited rural settings, where appropriate surgical techniques performed by qualified surgeons utilizing appropriate technologies and perioperative care teams can bring complex deformity care closer to the homes of many patients. This article was previously presented as a meeting abstract at the 2023 Society of Military Orthopaedic Surgeons (SOMOS) and the 2023 International Spinal Deformity Symposium (ISDS).

## 2. Materials and Methods

This is a retrospective, Institutional Review Board-approved study conducted at a 48-bed surgical specialty hospital on patients diagnosed with ASD and treated by a single surgeon. The surgical hospital does not have an on-site intensive care unit (ICU) but has transfer agreements in case of need. The radiological and clinical outcomes of all patients treated surgically with MIS approaches and the assistance of the ExcelsiusGPS^®^ robot (Globus Medical, Audubon, PA, USA) were collected prospectively in a database from July 2018 to May 2022. Retrospective review and analysis of this prospectively collected data resulted in the generation of the current study.

All consecutive adults with ASD who underwent robotic-assisted, minimally invasive primary or revision surgery were retrospectively reviewed. Inclusion criteria included the following: scoliosis with a Cobb angle ≥ 20, sagittal vertical alignment (SVA) ≥ 50 mm, or pelvic incidence (PI) and lumbar lordosis (LL) mismatch ≥ 10. MIS approaches with posterior fixation, including LLIF (L1–L4) or anterior lumbar interbody fusion (ALIF; L4–S1), were included. Exclusion criteria included the following: history of active infection at or adjacent to the surgical site, deformity caused by malignancy or severe metabolic bone disease, and fully open procedures.

### 2.1. Selection Criteria for Surgery

All patients were screened and cleared for surgery by an on-site hospitalist team based on the following optimization goals: hemoglobin A1C < 8.5%, body mass index (BMI) < 35, tobacco cessation, cardiac clearance, and bone density T-score > −2.5.

Patients with osteoporosis (T-score < −2.5) were evaluated by a local endocrinologist who specializes in bone health; these patients were placed on anabolic bone agents if not contraindicated for at least 3 months before surgery and continued for a year of total treatment. Tobacco cessation was encouraged through medically observed classes.

Obese patients (BMI > 35) were evaluated and counseled by a bariatric nutritionist to facilitate a weight loss plan and, when appropriate, were evaluated by a bariatric surgeon.

### 2.2. Surgical Technique

All procedures were performed using a two-stage approach conducted 1 day apart during a single hospital admission. The surgeries utilized a robotic-assisted workflow with preoperative computed tomography (CT) imaging for planning and navigation.

#### 2.2.1. First Stage

**Figure 1:**
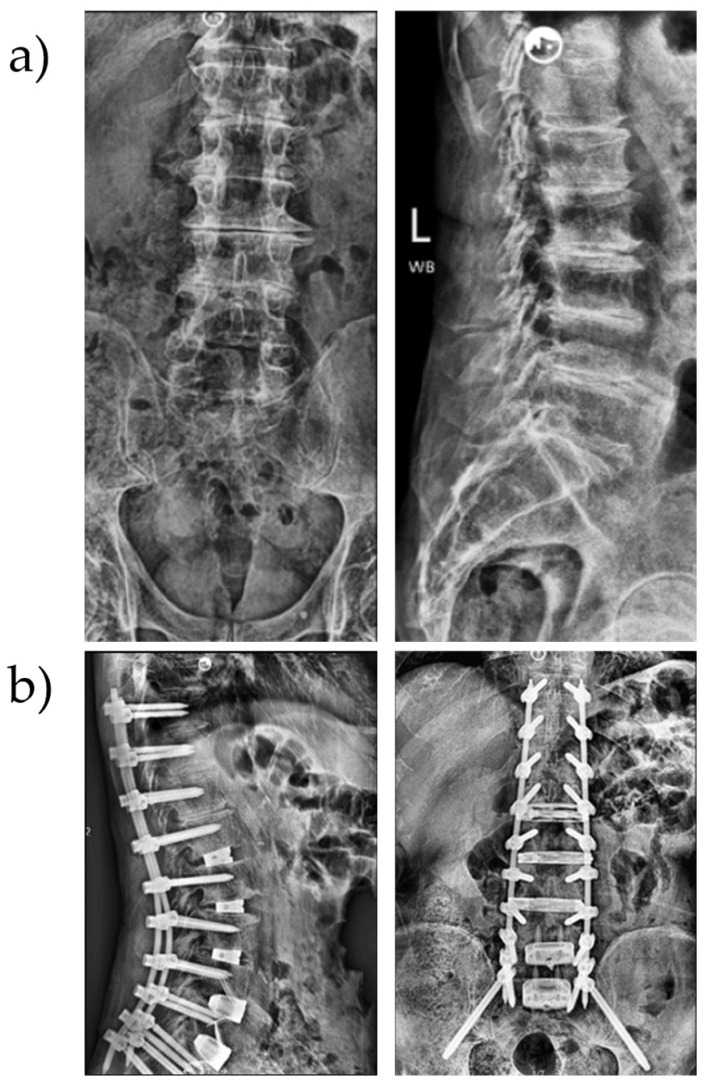
(**a**) Preoperative images of a patient with sagittal malalignment; (**b**) postoperative images showing expandable LLIF cages at L1–4, 3D-printed ALIF spacers at L4–S1 and robot-assisted posterior fixation from T10 to pelvis.

The first stage of the surgery was performed with the patient in the supine position. An access surgeon assisted in carrying out ALIF at the L4–S1 levels using a mini-open approach. Following completion of the ALIF, the patient was repositioned into the lateral decubitus position to facilitate the transpsoas LLIF from L1 or L2 to L4 (Figure 1). L1-2 was addressed through a lateral approach only when deemed necessary to aid in coronal or sagittal rebalancing.

#### 2.2.2. Between Stages

Immediately following the first-stage procedure, patients were transported directly from the post-anesthesia care unit (PACU) to the CT scanner. A postoperative CT scan was obtained to evaluate the placement of the anterior interbody spacers and to facilitate detailed planning of the posterior construct. Based on the CT scout images, the surgical team assessed the patient’s spinal alignment and determined the necessity for additional mini-open posterior osteotomies.

In addition, a focused physical examination was performed to assess changes in lower extremity radicular symptoms. The resolution or persistence of these symptoms guided the decision-making regarding whether indirect decompression achieved adequate neural element relief or whether mini-open direct decompression would be required during the second stage.

#### 2.2.3. Second Stage

Unless contraindicated, patients received a tranexamic acid (TXA) protocol, including a 1 g loading dose administered before the surgical incision, followed by a continuous infusion of a second 1 g dose over an 8 h period.

A midline skin incision of appropriate length was made and carried down to the level of the fascia. The fascia was dissected bilaterally to create access for transfascial screw placement via small stab incisions. A separate midline fascial incision was then made, extending from the planned uppermost instrumented vertebra (UIV) down to the L1 or L2 level (depending on the uppermost level with anterior column support). The spinous processes and medial laminae were decorticated, and bone graft material was placed over the prepared bony surfaces. The fascial pocket was subsequently closed, allowing for posterior bone grafting across all non-interbody levels using a mini-open approach.

Following graft placement, robotic registration was performed. Posterior stabilization was achieved with pedicle screw and rod constructs placed using robotic-assisted navigation based on the preoperative CT-based plan. Mini-open direct decompressions and posterior osteotomies were performed through small, targeted fascial incisions to optimize deformity correction and relieve neural compression if necessary. In cases where sacroiliac (SI) joint fusion was required, it was carried out during this second stage of surgery.

Upon completion of all instrumentation and decompression procedures, the fascial incisions were closed. The superficial layers were re-approximated to the midline, and a superficial drain was placed before final layered skin closure.

### 2.3. Data Collection

Patient variables, such as demographics, comorbidities, and American Society of Anesthesiologists (ASA) classification, were collected [17]. Surgical data obtained included operative time, estimated blood loss (EBL), length of stay (LOS), and levels instrumented. Patient-reported outcomes, including the Oswestry Disability Index (ODI) [18] and numeric rating scale (NRS) [19], were reported. Postoperative complications and return to the operating room were also documented. Spinopelvic radiographic parameters, including pelvic tilt (PT), pelvic incidence (PI), and lumbar lordosis mismatch (PI-LL), were assessed. Global sagittal and coronal alignment parameters assessed included the SVA of C7–S1 and the coronal vertical axis of the central sacral pelvic line (CSPL) to the central sacral vertical line (CSVL). Patients were routinely followed up at 3, 6, 12 and 24 months postoperatively. All radiographic measurements were performed by independent observers using Surgimap (Nemaris Inc., New York, NY, USA) on preoperative and postoperative standing full-length radiographs, following standardized techniques.

### 2.4. Statistical Analysis

Data were analyzed using the SPSS v20.0.0 software (IBM Corp., Armonk, NY, USA). Continuous interval data were presented as mean ± standard deviation (SD) and 95% confidence interval (95% CI), where applicable, with the significance level at 0.05. The Shapiro–Wilk test was used to assess the normality of the distribution of differences between preoperative and postoperative values for each continuous outcome variable; normality was confirmed for all primary endpoints, supporting the use of parametric testing. The paired Student’s *t*-test was used to compare preoperative and postoperative measurements, as each patient served as his or her own control in this repeated-measures design. The chi-squared test was used to compare categorical data frequencies. Given the limited number of pre-specified, independently hypothesis-driven comparisons—each addressing a distinct clinical outcome (radiographic parameters, ODI, and NRS)—a formal correction for multiple comparisons was not applied; individual *p*-values are reported for each comparison to allow for transparent interpretation of the results.

## 3. Results

There were 50 consecutive patients who satisfied the inclusion and exclusion criteria for analysis, with an average age of 70 years (range: 49–81); 64% were female (32/50). The mean BMI for the cohort was 30 kg/m^2^ (range: 18.7–42.2 kg/m^2^; Table 1). Thirty patients (60%) were nonsmokers, and the rest were either active or previous smokers. Based on ASA classification, 29 patients (59.2%) were class 2, and 20 patients (40.8%) were class 3. There were nine primary cases; the remaining (41 patients) were revision or secondary procedures. Twenty-three patients (46%) had a marked mismatch (>20°) between PI and LL (++ in SRS-Schwab classification sagittal modifiers [20]), and eight patients (16%) had PT and SVA greater than 30° and 9.5 cm, respectively (++ in SRS-Schwab classification sagittal modifiers).

Three interbody spacers and 16 screws were inserted on average into each patient during the first and second stages, respectively. A total of 78 ALIF and 80 LLIF spacers were placed. On average, eight spinal levels were instrumented for each patient. The average blood loss values for the first and second stages (robotic-assisted screw placement) were 62 mL and 205 mL, respectively. The average operative time for the combined procedure was 382 min (6.4 h), with 172 min accounting for the first stage and 210 min for the second stage (Table 1).

The average LOS in the hospital and follow-up period was 5 days and 10.6 months, respectively. No patients required a transfer to a facility with intensive care capabilities, and none required revision of hardware placement. Thirty-one patients (62%) were discharged home after the surgery, and the remaining were sent to an extended care facility or rehabilitation center.

There was an average reduction in the lumbar coronal scoliotic curve of 14.5° (baseline: 22.6°, final follow-up: 7.5°; 95% CI: 11.5–17.6) and an increase in lumbar lordosis of 14.8° (baseline: 42.3°, final follow-up: 57.0°; 95% CI: 11.4–18.1; *p* < 0.01). The average differences between PI and LL preoperatively and at the final follow-up were 17.6° and 9.6°, respectively (*p* < 0.01). The mean improvement in global SVA was 45.7 mm (95% CI: 5.7–85.7 mm; *p* = 0.03; Table 2).

Mean ODI (%) and NRS scores significantly improved by 33.8% (46.7 ± 13.3 to 30.9 ± 19.8; *p* < 0.01) and 55% (6.0 ± 2.2 to 2.7 ± 2.6; *p* < 0.01) at final follow-up (Table 3).

Four patients (8%) needed revision and extension of fusion because of adjacent segment disease. One patient (2%) developed retrograde ejaculation after the surgery, which improved spontaneously. Two patients (4%) were found to have proximal junctional kyphosis months following surgery, and one ultimately underwent revision and extension of fusion to T2 for proximal junctional failure. Two patients (4%) had a neurologic deficit following surgery. Three patients (6%) developed pneumothorax following surgery, which was managed successfully with insertion of chest tubes at the time of the anterior column reconstruction. One patient died of COVID-19-induced respiratory failure 17 months after the surgery.

## 4. Discussion

The main goal of ASD corrective surgery is to improve spinal alignment, relieve patients’ symptoms by appropriate decompression of neural elements, and achieve bony fusion of the realigned spine [21]. Open deformity correction is a major procedure with frequent perioperative complications [22]. MIS techniques can be used in spinal surgery to reduce blood loss, lower the complication rate, and shorten the operative time and may be more cost-effective compared to traditional open techniques [23]. Robotic assistance is an important adjunct in posterior stabilization of complex deformity correction, allowing for tissue-friendly surgical techniques without the need to increase radiation exposure, as is associated with traditional MIS surgery [24]. Although the argument for robotic applications in spine surgery has largely focused on safety, accuracy, and efficiency, this study examines this technology from a different perspective: its ability to complement complex ASD correction in a smaller surgical specialty hospital with limited resources compared to traditional large academic centers. While favorable perioperative and radiographic outcomes of MIS ASD surgery have been reported in the literature, these data have predominantly originated from large academic medical centers and multicenter consortia. It remains an open and clinically important question whether such outcomes can be reliably reproduced in smaller, non-academic settings. The primary purpose of the current study is, therefore, twofold: first, to demonstrate that the results traditionally reported by high-volume academic centers are reproducible within a single community-based surgical specialty hospital and, second, to specifically characterize the perioperative safety profile of this MIS robotic-assisted approach in a resource-limited environment.

The implementation of robotic-assisted deformity correction at this resource-limited surgical specialty hospital improved reliance on MIS techniques in deformity surgery to successfully achieve surgical goals with significant correction of spinal malalignment, improvement of patient-reported outcomes, and a low complication rate (24%), all of which were manageable at the present institution. Critically, the perioperative safety data underscore the feasibility of performing these complex procedures outside of a traditional academic center: no patients required transfer to a facility with ICU capabilities, no hardware revisions were necessary, estimated blood loss was markedly lower than historical open controls (mean total 267 mL), the average length of hospital stay was only 5 days, and the majority of patients (62%) were discharged directly home. These safety metrics are particularly noteworthy given that this 48-bed surgical specialty hospital does not have on-site intensive care capabilities, and they collectively demonstrate that an appropriately trained surgeon utilizing modern MIS and robotic-assisted techniques, supported by an optimized perioperative care protocol and dedicated care team, can achieve a safety profile comparable to that reported by large academic institutions.

Relevant historical benchmarks for open surgery can be found in the ASD correction literature. Daubs et al. published the outcomes and complications of ASD correction surgery using open techniques, which revealed an average blood loss of 2056 mL, a mean LOS of 13 days, and an average operative time of 10 h [25]. The results of a multicenter study comparing open versus MIS corrective surgery reported an average blood loss and surgical time of 1360 mL and 356 min (approximately 6 h), respectively, for open cases [26]. Finally, a retrospective study of patients treated with MIS transpsoas LLIF followed by an open posterior fixation within 5–7 days showed an average blood loss of 1966 mL, LOS of 20 days, and operative time of 7.3 h [27]. These outcomes are in sharp contrast to the present study’s results, in which the mean total blood loss, LOS, and operative time for the total procedures were 267 mL, 5 days and 6.4 h, respectively. In the current study, the surgeon used MIS techniques with navigation and robotic-assisted placement of pedicle screws for the posterior stabilization stage of the ASD reconstruction instead of open techniques. These differences may meaningfully contribute to the reduced operative time and blood loss reported in the present study.

In a retrospective study of non-robotic MIS deformity correction surgery, the average blood loss values for anterior and posterior procedures were 241 and 231 mL, respectively. The mean operating time was 232 min for the anterior procedures and 248 min for posterior stabilization. Mean LOS in the hospital was 10 days. Preoperative coronal Cobb angles, on average, were 22°, which corrected to 7° postoperatively [28]. These results are roughly comparable to the current study results except for the considerable difference in average length of stay (10 vs. 5 days). The difference may be due to a longer interval (3 days vs. one day) between the staged operations in that study. We found that performing the second stage 1 day after the first is beneficial for the patient because they tend to reach the physiological nadir of hemoglobin and hematocrit on or about postoperative day 3, which we believe to be the dilutional effect of IV fluids administered intraoperatively. As a result, performing surgery on postoperative day 3 is a physiologically disadvantageous time compared to the first postoperative day.

It is important to note that the present results should be interpreted in the context of a retrospective study and a single surgeon at one site, with a small sample size of 50 patients, as this may affect the generalizability of the results. Additionally, this study lacks a concurrent control group, as all patients treated at our institution during the study period underwent the same MIS robotic-assisted approach; no concurrent open surgery or non-robotic MIS cohort was available for direct comparison. The study, therefore, relies on comparisons with historical controls from the published literature to contextualize the results, and such comparisons are inherently limited by differences in patient populations, surgical techniques, institutional protocols, and era of treatment. Future prospective, comparative studies—ideally multicenter in design—would be valuable to more rigorously evaluate the relative advantages of robotic-assisted MIS ASD correction against other surgical approaches.

It should also be noted that the study period represents the learning curve associated with the use and adoption of the robotic system in deformity patients, and many of the inefficiencies in the early period have been improved with increased exposure to the platform. A limitation of this study is the lack of postoperative CT images for the entire cohort to precisely assess the accuracy of the inserted pedicle screws and fusion of instrumentation. However, the low rate of complications and the absence of any clinically evident hardware-related symptoms related to misplaced screws or pseudoarthrosis strongly suggest successful fusion and that the pedicle screws were inserted with a high level of accuracy.

Another limitation of this study is the relatively short follow-up period (mean 10.6 months), which hindered the author’s ability to evaluate potential long-term complications. Specifically, the current follow-up duration precludes a definitive assessment of important long-term outcomes such as pseudarthrosis, implant failure, late proximal junctional kyphosis, and adjacent segment disease—all of which are recognized complications of ASD corrective surgery that may manifest well beyond the early postoperative period and require extended surveillance to fully characterize. While the early complication data and radiographic outcomes presented herein are encouraging, the true durability of the correction and the long-term safety profile of this approach cannot be established without follow-up of at least 2 years. We attribute the relatively short follow-up to the disruption of normal postoperative protocols due to COVID-19 restrictions during the worldwide pandemic, which significantly impacted patients’ ability to return for scheduled follow-up visits during the study period. Future research with longer follow-up is needed to investigate the long-term success and complications of MIS robotic-assisted ASD corrective surgery, including rates of pseudarthrosis, hardware failure, proximal junctional kyphosis, and adjacent segment disease, and whether the results reported in this study can be exported to other facilities of similar size. Emerging non-invasive and non-irradiating measurement technologies, such as wearables or 3D posturography, may prove beneficial for improving follow-up compliance and creating new insights into long-term outcomes [29,30].

Additionally, research should concentrate on the cost-efficiency associated with shorter operative times; decreased EBL, LOS, and reoperation rates; less reliance on complex postoperative care environments such as the ICU; and an increased number of patients discharged to home as opposed to long-term care facilities. Such research may demonstrate considerable savings in the cost of health care associated with robotic ASD correction and may support the increased benefit of providing these services to our aging population at more centers across the United States by qualified surgeons with dedicated operating rooms and patient care teams.

## 5. Conclusions

The current study demonstrated the perioperative efficacy of MIS robotic-assisted ASD correction at a small surgical specialty hospital with successful correction of sagittal and coronal imbalances in moderate deformities. This study outlines a formula for perioperative patient care and a tissue-friendly surgical approach to deformity correction, resulting in significantly reduced hospital LOS, EBL, and operative times compared to historical data performed with open procedures. This allows these complex cases to be done at smaller facilities with limited resources.

## Figures and Tables

**Table 1:** Demographic and surgical data.

Characteristic	Total *N* = 50
**Mean age (SD) (range) (years)**	70 (6.4) (49–81)
**Mean BMI (SD) (range) (kg/m^2^)**	30 (5.2) (18.7–42.2)
**Sex, *n* (%)**	
Female	32 (64.0)
Male	18 (36.0)
**Smoking status, *n* (%)**	
Nonsmoker	30 (60.0)
Active or previous smoker	20 (40.0)
**ASA classification, *n* (%)**	
Class 2	29 (58.0)
Class 3	20 (40.0)
Not available	1 (2.0)
**Primary cases, *n* (%)**	9 (18.0)
**Secondary/revision cases, *n* (%)**	41 (82.0)
**Sagittal parameter mismatch, *n* (%)**	
PI–LL mismatch > 20°	23 (46.0)
PT > 30° and SVA > 9.5 cm	8 (16.0)
Other	19 (38.0)
**Mean estimated blood loss (SD) (range) (mL)**	
First stage	62 (61.2) (5–300)
Second stage	205 (210.8) (20–900)
**Mean operative time (SD) (range) (minutes)**	
First stage	172 (63.8) (45–391)
Second stage	210 (91.0) (50–454)
**Combined procedure, minutes (hours)**	382 (6.4)
**Mean length of hospital stay (SD) (range) (days)**	5 (3.6) (2–25)

**BMI** = body mass index; **ASA** = American Society of Anesthesiologists; **PI** = pelvic incidence; **LL** = lumbar lordosis; **PT** = pelvic tilt; **SVA** = sagittal vertical alignment.

**Table 2:** Changes in radiographic parameters (mean ± standard deviation).

Radiographic Parameter	Preoperative	Latest Follow-Up	Change	*p*-Value
**Major Scoliotic curve**	22.6° ± 14.5°	7.5° ± 7.4°	−14.5° ± 10.5°	<0.01 *
**LL**	42.3° ± 15.6°	57.0° ± 14.5°	14.8° ± 11.7°	<0.01 *
**PI-LL**	17.6° ± 12.0°	9.6° ± 8.0°	−8.0° ± 12.5°	<0.01 *
**Pelvic Tilt**	25.1° ± 7.6°	22.0° ± 7.5°	−3.0 ± 6.8°	0.04 *
**SVA (mm)**	85.4 ± 62.6°	39.7 ± 30.4	−45.7 ± 52.0°	0.03 *

**PI** = pelvic incidence; **LL** = lumbar lordosis; **PT** = pelvic tilt; **SVA** = sagittal vertical alignment. * Statistically significant (*p* < 0.05) using Student’s *t*-test.

**Table 3:** Changes in patient-reported outcomes (mean ± standard deviation).

Patient Reported Outcome	Preoperative	Latest Follow-Up	Change (%)	*p*-Value
**ODI**	46.7 ± 13.3	30.9 ± 19.8	33.8	<0.01 *
**NRS**	6.0 ± 2.2	2.7 ± 2.6	55	<0.01 *

**ODI** = Oswestry Disability Index; **NRS** = numeric rating scale. * Statistically significant (*p* < 0.05) using Student’s *t*-test.

## Data Availability

All data is maintained at and available upon request from the Northwest Specialty Hospital Research Department.

## Abbreviations

The following abbreviations are used in this manuscript:

ASD	Adult spinal deformity
MIS	Minimally invasive surgical
LLIF	Lateral lumbar interbody fusion
ICU	intensive care unit
SVA	Sagittal vertical alignment
PI	Pelvic incidence
LL	Lumbar lordosis
ALIF	Anterior lumbar interbody fusion
BMI	Body mass index
CT	Computed tomography
PACU	Post-anesthesia care unit
TXA	Tranexamic acid
UIV	Uppermost instrumented vertebra
SI	Sacroiliac
ASA	American Society of Anesthesiologists
EBL	Estimated blood loss
LOS	Length of stay
ODI	Oswestry Disability Index
NRS	Numeric rating scale
PT	Pelvic tilt
CSPL	Central sacral pelvic line
CSVL	Central sacral vertical line
SD	Standard deviation
CI	Confidence interval
